# Psychotic-like Experiences and Underlying Mechanisms: An Integrative Model of ADHD Symptoms, Rumination, Negative Affect, and Trauma Experience

**DOI:** 10.3390/jcm13226727

**Published:** 2024-11-08

**Authors:** Hanna Gelner, Paulina Bagrowska, Bertus F. Jeronimus, Błażej Misiak, Jerzy Samochowiec, Łukasz Gawęda

**Affiliations:** 1 Experimental Psychopathology Lab, Institute of Psychology, Polish Academy of Sciences, Stefana Jaracza 1, 00-378 Warsaw, Poland; pbagrowska@psych.pan.pl (P.B.); l.gaweda@psych.pan.pl (Ł.G.); 2 Department of Psychology, University of Groningen, 9712 CP Groningen, The Netherlands; b.f.jeronimus@rug.nl; 3 Department of Psychiatry, Wroclaw Medical University, 50-367 Wroclaw, Poland; blazej.misiak@umed.wroc.pl; 4 Department of Psychiatry, Pomeranian Medical University, 70-204 Szczecin, Poland; jerzysamochowiec@gmail.com

**Keywords:** psychosis, abuse, neurodevelopmental changes, negative repetitive thinking, negative emotions

## Abstract

**Background:** Psychotic-like experiences (PLEs) are low-intensity subclinical phenomena, often transient in nature. The etiology of PLEs primarily involves neurodevelopmental changes, trauma exposure, and maladaptive coping styles. Attention-Deficit/Hyperactivity Disorder (ADHD) is considered to be one of the factors that increase the risk of future psychosis. Furthermore, ADHD symptoms predict a heightened incidence of traumatic experiences, ruminative thoughts, and negative affect (NA). This present study examines whether rumination and NA mediate the relationship between ADHD symptoms and PLEs and whether trauma experiences moderate these pathways. **Methods:** A total of 188 participants (72% female) aged 18–35 completed questionnaires assessing ADHD symptoms and traumatic experiences and took part in a seven-day experience sampling method (ESM) procedure, completing ratings of PLEs experiences, the intensity of ruminations, and NA. **Results:** Correlation analysis showed significant relationships between all tested variables. Serial mediation analysis revealed a significant indirect effect of rumination and NA in the link between ADHD symptoms and PLEs. There was no significant impact of trauma experience in this relationship. **Conclusions:** Our study underscores the important role of rumination and NA in the co-development of ADHD symptoms and PLEs. Future research should consider investigating the intra-individual dynamics of ADHD and trauma using ecologically valid research methods in the context of PLEs to better understand these complex relationships.

## 1. Introduction

Psychotic-like experiences (PLEs) refer to hallucinations or delusions defined as subclinical phenomena, encompassing both delusion-like experiences and perceptual abnormalities [1,2] with a limited intensity, transience, or functional impairment. They are situated at the lower end of the psychosis continuum [1], representing a category of low-grade positive psychotic symptoms that do not meet the criteria for a psychosis spectrum disorder [3,4]. Because PLEs are far more common than severe psychotic symptoms in the clinical context [5] and affect approximately 5–7% of adults in the general population [6], their impact and associated costs may be still important [7]. Individuals with PLEs report more distress [8], more depressive symptoms [9], lower general functioning [8], and increased suicide risk [10,11], among others, compared to healthy controls. While PLEs typically resolve naturally over time in most individuals (~80%), a significant minority (∼7%) progresses into full-blown psychosis [12]. Despite a growing interest in PLEs over the past two decades, the mechanisms underlying hallucinations/delusions remain poorly understood. Research suggests that neurodevelopmental changes [13] and the experience of (early childhood) trauma are potential mechanisms of the risk of psychosis and its different stages (e.g., PLEs, full-blown psychosis; see [14,15,16]. Also, the use of maladaptive coping strategies, such as ruminative thought and the experience of negative affect, have been shown to increase the risk of PLEs occurrence [17,18]. An integrative theoretical model that links early developmental changes, trauma experience, and coping strategies in the context of PLEs remains necessary. Therefore, this present study focuses on the role of Attention-Deficit/Hyperactivity Disorder (ADHD) symptoms on PLE occurrences, as ADHD is a proxy for neurodevelopmental disadvantage (given the wide variety of associated neurological disorders; see [19]. The objective of this study is to test whether the association between the severity of ADHD symptoms and PLEs is mediated by the potential role of rumination and negative affect in daily life, and if the reported traumatic experiences strengthen this relationship. Hence, we examine whether (a) ADHD symptoms predict more PLEs, whether (b) rumination and negative affect mediate this association, and whether (c) the traumatic experiences over the life course moderate these processes.

### 1.1. The Role of ADHD and Trauma in the Context of PLEs

The sociodevelopmental–cognitive model of psychosis posits that schizophrenia is a neurodevelopmental disorder [20], which may explain the covariance with other neurodevelopmental disorders, especially Attention-Deficit/Hyperactivity Disorder (ADHD; [21,22]). ADHD symptoms are strongly associated with PLEs [23], and children diagnosed with ADHD exhibit a fivefold increased risk of developing psychotic disorders [24,25]. A systematic review of 15 studies and a meta-analysis of 12 studies conducted by Nourredine et al. (2021) [25] showed that up to 13% of patients diagnosed with schizophrenia were also diagnosed with ADHD, while adult ADHD symptom severity was associated with more paranoid thoughts and auditory hallucinations [26]. Finally, around 80% of schizophrenia patients exhibit a progressive cognitive decline from early adolescence, leading to functional disability and secondary (indirect) illness costs [27,28].

The sociodevelopmental–cognitive model of psychosis emphasizes the role of adverse childhood experiences on gene expression and dopaminergic dysregulation [13], resulting in abnormal stimulus processing, including the salience dysregulation that marks the psychosis spectrum [29,30]. Such neurodevelopmental changes arise from interactions between the environment, genes, and traumatic/adverse experiences, and dopaminergic dysregulation may result in abnormal stimulus processing, paranoid interpretations, and psychotic experiences, a process exacerbated by dysfunctional cognitive schemas [13]. Consequently, individuals with maladaptive cognitive biases are more susceptible to experiencing stress, paranoia, and/or hallucinatory experiences, potentially perpetuating or worsening psychotic beliefs.

Neurodevelopmental theories are supported by the threefold increase in the risk of developing psychotic disorders in individuals who experienced childhood trauma [31]. Childhood trauma may intensify the impact of social stress during adolescence and young adulthood when the risk of developing psychosis increases and the hypothalamic–pituitary–adrenal (HPA) axis becomes overactive or dysregulated [32]. Consequently, individuals at risk of psychosis demonstrate a heightened sensitivity to daily stressors and alterations in HPA functioning [33,34]. Furthermore, research has demonstrated that trauma is associated with an elevated risk of psychosis through the formation of cognitive biases [9,35], which in turn may render victimized people more susceptible to stress, paranoia, and/or hallucinatory experiences, and perpetuate or even exacerbate their psychotic beliefs. 

### 1.2. The Role of Rumination Thoughts and Negative Affect in the Context of PLEs

Emotional dysregulation is a key symptom of ADHD [36] and is evidenced by deficits in self-regulation [37,38]. Self-regulation deficits are evident in low frustration tolerance and high mood lability [39]. Indeed, individuals diagnosed with ADHD are more likely to report negative emotions, including anger, irritability, and frustration, and to ruminate more [40,41,42], thus causing persistent thoughts and mental images focusing on negative emotions and symptoms, pondering their causes, meanings, and consequences [43,44]. These symptoms have recently been proposed as one of the primary symptoms of ADHD [45]. Additionally, ADHD-related symptoms indirectly exacerbate depressive and anxiety symptoms through ruminative thoughts, significantly impacting an individual’s functionality [43]. 

The experience of distress or anxiety can elicit intrusive thoughts and more negative emotions [46]. Rumination frequency predicts anxiety and depression symptom severity [44,47] and more negative emotional outcomes and distress from psychosis symptoms [48,49]. Negative emotions play a pivotal role in the emergence of psychotic content and/or symptoms and underlie all emotional and psychotic disorders [50,51]. Such negative emotions are thought to be induced by meta-cognitions (“thinking about thinking”), meta-beliefs, and poor coping that amplify psychological distress [52]. Wells and Matthew’s model (1994, 1996) [53,54] on metacognitions focuses on maladaptive coping strategies and metacognitive beliefs that contribute to psychological distress by inducing negative emotions. For example, ruminative thinking, a maladaptive coping mechanism, likely plays an important role in the distressing experience of psychosis. A review reported more rumination in patients with psychosis than in healthy controls [18] and poorer self-regulatory strategies among schizophrenia patients than in the controls [55]. Furthermore, rumination or repetitive negative thinking is more common among adults who have lived through childhood adversity [56,57,58]. 

Ruminative thoughts can focus attention on threatening stimuli and create false associations between unrelated events and thoughts (salience dysregulation), which in turn perpetuates ruminative thoughts associated with delusions and more negative emotions [59]. Additionally, re-experiencing traumatic events and using maladaptive coping strategies to regulate emotions may contribute to experiencing auditory or visual hallucinations or persecutory imagery [60,61,62]. 

Despite the existence of established links between ADHD symptoms, ruminations, negative affect, trauma, and psychotic-like experiences, their interactions and codependencies remain unclear. Therefore, this present study aims to address this gap in the literature by examining whether ruminations and negative affect mediate the link between ADHD and PLEs and whether the trauma experience moderates these mediation pathways. 

### 1.3. This Study

This present study is focused on the role of ADHD symptoms in the occurrence of PLEs. Our aim is to determine whether the relationship between ADHD and PLEs is mediated by rumination and negative affect experienced in daily life and whether this connection is exacerbated by reported traumatic events. Hence, we examine three key questions: (a) whether ADHD symptoms predict more PLEs, (b) whether the ADHD–PLE relationship is mediated by rumination and negative affect, and (c) whether these effects are moderated by differential exposure to traumatic events through life. This resulted in the following hypotheses:

**H1.**  *ADHD symptoms predict more PLEs.*

**H2.**  *Rumination and negative affect mediate the relationship between ADHD symptoms and PLEs (serial mediation).*

**H3.**  *The link between ADHD symptoms and PLEs (**H1** + **H2**) is moderated by exposure to traumatic life events, such that the positive relationship between ADHD symptoms and PLEs through rumination and negative affect will become stronger in people who developed under conditions of higher levels of traumatic experience (moderated serial mediation).*

## 2. Methods and Materials

### 2.1. Participants

Data were derived from 188 participants (including participants in the control group N = 89 and participants in the experimental group N = 99) of a community sample recruited in three Polish cities (i.e., Warsaw, Wroclaw, Szczecin) for an ongoing study into epigenetic processes and associations with momentary stressors and psychotic-like experiences.

Included participants were (1) aged 18–35 years, (2) without a history of psychiatric treatment, and (3) with psychotic-like symptoms (PLEs) based on 16 items presented in the Recruitment Phase below, a global rating of 3–4 points for reference imagery and/or suspicion and persecutory imagery *(e.g., “I often have the feeling that other people are against me.”, “I was worried about being stalked.”, “I spend my time thinking that my friends are gossiping about me.”)*, and/or a global score of 3–4 points for hallucinations *(e.g., “I hear a voice speaking my thoughts aloud.”, “I can hear things that other people can’t hear, such as the voices of people who are whispering or talking.”, “I can see things that other people can’t see.”).* Participants who scored between 48 and 64 points were assigned to the experimental group, and those with 0–32 points became the control group (a global rating of 0–2 points for reference imagery and/or suspicion and persecutory imagery and/or a global score of 0–2 points for hallucinations). Exclusion criteria included (1) a lifetime history of psychiatric treatment, (2) a current episode of major depressive disorder (MDD), and/or (3) a diagnosis of substance use disorder (other than nicotine dependence).

The study protocol was approved by the Ethics Committees of the Institute of Psychology (Polish Academy of Sciences, Warsaw, Poland, approval number: 16/VII/2022), Wroclaw Medical University (Wroclaw, Poland, approval number: 129/2022), and Pomeranian Medical University (Szczecin, Poland, approval number: KB-006/25/2022).

### 2.2. Materials

The participants were required to complete a seven-day experience-sampling method (ESM) procedure, a clinical interview comprising a comprehensive Mini-International Neuropsychiatric Interview (M.I.N.I.), and the Comprehensive Assessment of At-Risk Mental States (CAARMS) (see Supplementary Tables S1 and S2 for prevalence scores on M.I.N.I. and CAARMS scales in the study sample). Furthermore, the participants were required to complete a series of questionnaires, including the Traumatic Experience Checklist (TEC; [63]), all described in detail below.

Demographics. Participants were asked to self-report their age, gender, educational level, and family history of psychiatric illness (i.e., depression and schizophrenia spectrum disorders).

The Comprehensive Assessment of At-Risk Mental States (CAARMS; [64,65]) is a semi-structured interview to determine ultra-high risk (UHR) status and measure a range of subthreshold symptoms associated with the prodromal phase of psychotic disorders. The CAARMS provides an intensity and frequency score for each item. It consists of 27 items grouped into seven scales: positive symptoms, cognitive changes, emotional disturbance, negative symptoms, behavioral changes, motor/physical changes, and general psychopathology. The scores for each of the subscales range from 0 to 6. The CAARMS was selected for this study due to its demonstrated efficacy in identifying individuals at ultra-high risk (UHR) for psychosis, exhibiting superior predictive accuracy and flexibility compared to other diagnostic instruments, as evidenced by the findings of Wang et al. (2022) [66]. 

The Mini-International Neuropsychiatric Interview (M.I.N.I.; [67]) was developed to assess the diagnoses of psychiatric patients according to DSM-IV and ICD-10 criteria. In order to verify the diagnoses, the gold standard was applied through the use of a structured diagnostic interview M.I.N.I. All the items in the questionnaire are answered with a ‘Yes’ or ‘No’ answer, starting with the screening questions and ending with the diagnostic blocks to check whether a patient meets the diagnostic criteria. In this study, the Polish version of M.I.N.I. Plus 5.0.0 was used, including all the modules. 

The Adult ADHD Self-Report Scale (ASRS; [68]) is an 18-item self-report screening scale for adult Attention-Deficit/Hyperactivity Disorder (ADHD). Responses range between 0—“never” and 4—“very often”. The total score ranges between 0 and 72. The Symptom Checklist is an instrument consisting of the eighteen DSM-IV-TR criteria. Six of the eighteen questions from part A (1–6) are the most predictive of symptoms consistent with ADHD. For part A, points are summed for a range of 0–24, with a cut-point ≥ 14 for ADHD. The total score can be classified into four categories: 0–9 is low negative, 10–13 is high negative, 14–17 is low positive range, and 18–24 is high positive range. The remaining twelve questions (7–18) are included in part B of the Symptom Checklist. The Cronbach’s alpha of the ASRS was 0.92 in our sample. The Adult ADHD Self-Report Scale (ASRS) was selected on the basis of its demonstrated reliability and validity in large-scale, cross-cultural studies, which have effectively captured ADHD symptoms in diverse populations (see [69]).

The Traumatic Experiences Checklist (TEC; [63]) is a 29-item self-report questionnaire addressing potentially traumatizing serious emotional events categorized across three types of trauma, referred to by six subscales: (1) emotional trauma captures emotional neglect and/or emotional abuse in various social settings with six items (*e.g., “Emotional neglect (e.g., being left alone, not shown enough affection) by parents or siblings. Has this happened to you?”*); (2) sexual trauma captures sexual harassment and/or sexual abuse in various social settings with six items (*“Sexual harassment (acts of a sexual nature in which there is NO physical contact) by parents or siblings. Has this happened to you?”*); and (3) bodily threat, which captures physical abuse in various social settings and intentional threat to one’s life, bizarre punishment, or intense pain, with six items (*e.g., “Physical abuse (e.g., being hit, bullied or physically hurt) by parents or siblings. Has this happened to you?”*). Scale scores for emotional, sexual, and bodily trauma are calculated by summing the presence scores for the relevant items, with six items each for emotional and sexual trauma and bodily threat. The Cronbach’s alpha of the TEC total was 0.74 in our sample, and the subscales were as follows: emotional trauma 0.66, sexual trauma 0.31, and bodily trauma 0.39. The Trauma Exposure Checklist (TEC) was selected on the grounds of its status as a valid and reliable tool for assessing trauma exposure and its associated risk and protective factors. This is evidenced by its robust factorial structure and psychometric properties, as demonstrated in recent studies (e.g., [70]). The experience sampling method (ESM) questionnaires covered various domains of psychotic-like experiences (PLEs). All items included in the statements are presented in Table 1. While most of the items used in the questionnaires were adopted from previous studies [71,72,73], we also included items adapted from the PQ-16 [74] to capture the presence of PLEs. In the ESM procedure, participants also responded to questions regarding ruminative thoughts and negative affect (NA), as well as to additional questions that were not pertinent to this present study (see Supplementary Table S3). However, only items related to PLEs, ruminations, and NA are analyzed here. The order of items was not randomized. 

### 2.3. Procedure

#### 2.3.1. Phase I

The recruitment of study participants began with an extensive screening process using snowball sampling via social media and survey websites. We recruited 4203 participants for a web-based survey, who completed a 16-item screener to identify the presence of psychotic-like experiences (PLEs) in the previous month (between April and October 2022) derived from the following questionnaires: (1) the Revised Hallucination Scale (RHS; three items, [75,76,77]); (2) the Revised Green Paranoid Thoughts Scale (GPTS; four items, [78]); and (3) the Prodromal Questionnaire-16 (PQ-16; nine items, [74]). Participants were also asked to complete the 14 screening questions of the Mini-International Neuropsychiatric Interview (M.I.N.I.; [67]) for depression, mania, panic attacks, anxiety, agoraphobia, social phobia, obsessive–compulsive disorder (OCD), post-traumatic stress disorder (PTSD), and alcohol/drug abuse. Participants were also asked to fulfill the Adult Attention-Deficit/Hyperactivity Disorder (ADHD) Self-Report Scale (ASRS; [68]).

#### 2.3.2. Phase II

The second screening step included telephone interviews with participants with the highest PLEs scores, constituting the experimental group, and participants with the lowest PLEs scores, referred to as the control group. To provide clinical validation of the presence of current psychotic-like experiences, selected questions from the Comprehensive Assessment of At-Risk Mental States (CAARMS; [64,65]) were used, examining the following symptoms: (1) ideas of reference (*e.g., “Have you felt that things that were happening around you had a special meaning, or that people were trying to give you messages?”*), (2) suspiciousness and persecutory ideas (*e.g., “Do you feel like people have been talking about you, laughing at you, or watching you?”*), and (3) hallucinations (*e.g., “Do you ever hear things that other people seem not to, such as sounds, or voices?”*). Furthermore, individuals who tested positive for major depressive disorder (MDD) and substance use disorders underwent additional testing using the Mini-International Neuropsychiatric Interview (M.I.N.I.). Those who met the criteria for any of the aforementioned disorders were disqualified from participating in this study.

#### 2.3.3. Phase III

Participants who met all inclusion criteria were invited to a face-to-face diagnostic interview, were informed about the experience sampling method (ESM) procedure, and were provided with all necessary materials. Prior to the commencement of the interview and study procedure, participants were required to complete a series of documents, including an informed consent form. This document detailed the procedures and stages involved in this study, as well as the option to withdraw from the research at any point without providing a reason. It also outlined the remuneration offered, the requirements for obtaining it, and information regarding the processing of personal data, the anonymity of the research, and collective data analysis. 

#### 2.3.4. Experience Sampling Assessment

The ESM procedure was conducted over seven days, with six assessments per day (42 assessments in total) administered between 9 a.m. and 10 p.m. with a minimum 60 min gap between prompts (a stratified randomization strategy) via the MovisensXS application on provided smartphones, Version 1.5.23 (Movisens GmbH, Karlsruhe, Germany; https://www.movisens.com, accessed on 28 April 2024). Prior to the beginning of the protocol, participants were provided with detailed instructions and a handout with all relevant information. Participants were informed that they were required to respond to each beep directly or delay it for up to 15 min. Failure to comply with these instructions resulted in the survey being considered incomplete, which accounted for a total of 3.6% of all surveys. In order to encourage participation in the study, respondents who achieved a response rate of at least 80% were awarded a prize of EUR 250. The threshold was set in accordance with recommendations identified in previous studies in the field of experiential sampling methodology [79,80]. Accordingly, the decision was taken to set the minimum response rate in the ESM to 80% in order to ensure the reliability of the collected data, while allowing for the possibility of unforeseen occurrences. Consequently, the requirements were reduced from 100% to the aforementioned 80%. Participants who encountered technical difficulties or required further clarification were encouraged to establish contact with the experimenter via email or telephone. Once the experience-sampling period was complete, participants were invited to the final face-to-face meeting with the experimenter to receive their duly compensated remuneration. Participants were asked if any unusual events had occurred during the previous week and, if necessary, provided information about the availability of psychological support. 

### 2.4. Analysis

Statistical analyses were performed in SPSS 29. The analyses include all participants included in this study, without a distinction between the groups. It should be noted that the groups in this study were recruited for the main project. Nevertheless, the characteristics of the study groups are presented in the following section (see Table 2, Descriptives) and in the descriptive characteristics of the study subgroups (see Supplementary Tables S6 and S7). 

Pearson’s correlation analyses were conducted to investigate the relationships between ADHD, PLEs, ruminations, negative affect, general trauma experience, and three subtypes of trauma (emotional, sexual, and bodily threat trauma). We use correlations (*r*) and betas (β) as effect size indices to express our results, which we regard to be small if they are between 0.10 and 0.19, moderate between 0.20 and 0.29, and large from 0.30 based on normative effect sizes that are commonly found [81,82,83]. For this typical effect size of around *r* = 0.20, one study needs at least 150 participants but, ideally, up to 250 participants to reduce estimation error in correlations [84]. The medium effect size was based on Cohen’s (1988) [85] notion that it should be noticeable to the naked eye of a careful observer. We prefer practical significance (effect sizes) over statistical significance (*p*-values), which means we adhere to conventional *p*-values unadjusted for multiple testing) [85,86] and focus on effects significant at *p* < 0.05.

The strong correlations between several independent variables, including PLEs, rumination, and negative affect (ranging from 0.72 to 0.89, see Table 3), could indicate multicollinearity, which can indicate that variables are close to perfect linear combinations of one another, resulting in potentially unstable regression estimates and, thus, wide standard errors and unreliable significance tests [87]. When we examined the Variance Inflation Factors (VIFs, see Supplementary Table S4), they indicated salient but moderate inflation (all VIFs < 4.8, but close to 5, see [87]). When we examined the condition index (CI) of our correlation matrix (a function of their eigenvalue collinearity), however, there was no indication of variable collinearity problems (CI = 3.96 for the fourth and 4.81 for the fifth dimension; see Table S5 and following [87]). We, therefore, have reasonable trust in our multiple regression models estimated with robust confidence intervals, as described below.

Student’s *t*-test for independent samples was conducted to assess the group differences in outcomes between the female (N = 136) and male (N = 52) participants and for the experimental (N = 99) and control groups (N = 89) (see Supplementary Tables S6 and S7 for descriptive characteristics of the subgroups).

To test our hypotheses, serial mediation and moderated serial mediation analyses were conducted using the PROCESS macro for SPSS [88]. The dataset included variables from both ESM measures (PLEs, rumination, and negative affect) and self-report questionnaires (ADHD and trauma). The ESM data were aggregated in order to align with the other variables included in the model, which were measured at a single time point. This aggregation process involved averaging the measurements, including those obtained via ESM (negative affect, rumination, PLEs) and those collected at a single time point (ADHD symptoms, trauma) to create a single score that reflects the participants’ experiences over the entire time frame. This approach enabled the analysis of all variables, including those measured via ESM and those collected at a single time point, on an equivalent level for comparative and inferential purposes. For the ESM data, the results of those who achieved a response rate of at least 80% were included, and missing responses were not included in the calculated average of PLEs, negative affect, and rumination.

First, serial mediation analysis was used to investigate the role of ruminative thoughts (M_1_) and negative affect (M_2_) in the relationship before ADHD (X) and PLEs (Y), using Model 6 in the SPSS PROCESS macro. Secondly, general trauma experience scores and the three subscales of trauma separately, namely, emotional, sexual, and bodily trauma, were added as moderators of the mediation model, resulting in a moderated serial mediation analysis (using Model 85 in the PROCESS macro) to test whether trauma experience (W) would moderate the role of mediator ruminations (M_1_) and negative affect (M_2_) in the relationship between ADHD (X) and PLEs (Y). Third, we fit similar models separately for three subscales of trauma to explore whether any particular type of traumatic experience played a unique role in the model. We tested our hypothesis using 95% bootstrapped confidence intervals (CIs) in PROCESS, generated with 5000 bootstrapped samples.

## 3. Results

The study sample consisted of 72.3% female participants aged 18–35 (M = 25.21, SD = 5.18). Of these, 54.3% had completed higher education, while 42% had completed secondary education; see Table 2. The results for individual questionnaires and sample characteristics are presented in Table 2. The M.I.N.I. diagnoses are provided in Supplementary Table S1, and the CAARMS diagnoses are in Supplementary Table S2. Our sample comprised 61 individuals who met the study criteria of having an initial ADHD diagnosis according to the ASRS part A cut-off point, of which 53 individuals were in the experimental group. According to the M.I.N.I. diagnosis, 25 participants (13% total) met the criteria for ADHD (see Supplementary Table S1). The results of Student’s *t*-test for independent samples (see Supplementary Table S6) indicated that women exhibited significantly higher scores in ruminative thoughts over the week (*p* < 0.05, d = 0.39), higher values in negative affect over the week (*p* < 0.05, d = 0.46), and had higher total scores on the trauma questionnaire (*p* < 0.05, d = 0.35), with a particularly notable effect observed in one of the subscales, sexual trauma (*p* < 0.001, d = 0.65). With regards to the study group comparison (High × Low) (see Supplementary Table S7), the results indicated that the participants in the experimental group were younger (*p* < 0.001, d = −0.54) and exhibited significantly higher scores on the ASRS questionnaire for ADHD symptoms (*p* < 0.001, d = 1.76), as well as in part A for the predictive list of ADHD symptoms (*p* < 0.001, d = 1.40) and in part B for the control list of ADHD symptoms (*p* < 0.001, d = 1.70). Furthermore, the results indicated significantly higher scores in the experimental group in the weekly average of PLEs experiences (*p* < 0.001, d = 1.39), higher values in ruminative thoughts over the week (*p* < 0.001, d = 1.23) and negative affect (*p* < 0.001, d = 1.51), and higher scores on the total trauma experience questionnaire (*p* < 0.001, d = 0.95), and all its subscales, including emotional trauma (*p* < 0.001, d = 0.95), sexual trauma (*p* < 0.001, d = 0.69), and bodily threat (*p* < 0.001, d = 0.5). 

### 3.1. Power Analysis

The post hoc power analysis specified a sample size of 188 and an alpha level of *p* < 0.05, considering an effect size (*f*^2^) of 0.15 in the G*Power3 program [89], revealing a power of >0.99. The sample size was adequate for the study to detect medium-sized effects.

### 3.2. Correlations Analysis

We calculated correlations between the study variables, which are presented in Table 3. All variables included in the model showed a significant correlation (0.19–0.89, *p* < 0.001). Both ADHD and PLEs showed stronger covariance with rumination and negative affect (0.7) than trauma experiences (0.3). 

### 3.3. Serial Mediation

**Figure 1:**
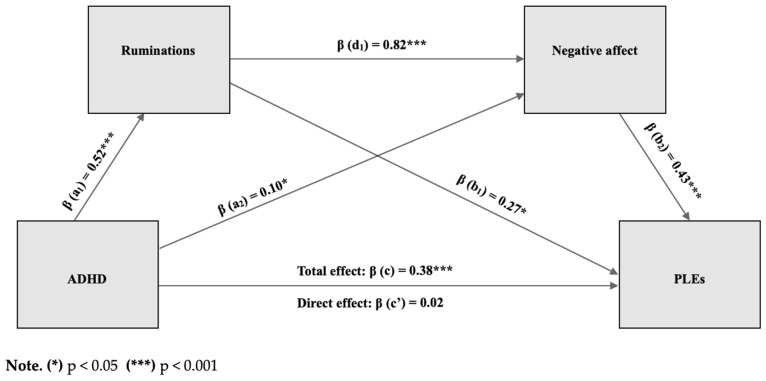
Serial mediation model (Model 6).

The hypotheses that ADHD symptoms predict PLEs (**H1**) in part through the role of ruminative thoughts and negative affect (**H2**) were tested with a serial mediation analysis using Model 6 in the PROCESS macro for SPSS ([90]; see Figure 1). The results showed that the standardized total effect of ADHD on PLEs was significantly different from zero (β = 0.38, *p* < 0.001, 95% CI = 0.24 to 0.51). The direct effect of ADHD on PLEs was not significant, indicating that mediation is indirect only [91] (β = 0.02, *p* = 0.81, 95% CI = -0.11 to 0.14). The total indirect effect of ADHD on PLEs was found to be significant (β = 0.36, 95% CI = 0.25 to 0.50), as well as through NA only (β = 0.04, 95% CI = 0.003 to 0.11) and through ruminations and negative emotions (β = 0.18, 95% CI = 0.05 to 0.34). The indirect effect through ruminations only was not significant (β = 0.14, 95% CI = −0.01 to 0.31). The total effect explained 18.7% of the variance in PLEs, and the serial mediation model explained 49.5% of the variance in PLEs. Gender and age were added to the model as covariates but turned out not to be significant predictors.

### 3.4. Moderated Serial Mediation

Subsequently, to test our last hypothesis (**H3**) that traumatic experiences would moderate the mediation effects, we conducted a moderated serial mediation (see Figure 1) using Model 85 in the PROCESS macro for SPSS [90], in which we added trauma experience as a moderator (W) to our model. This moderated serial mediation pathway model did not support the idea that participants with ADHD who reported more adverse (traumatic) experiences were more vulnerable to PLEs because they ruminate more and report more negative emotions. The trauma scale did not moderate any of the mediation pathways (see Table 4). Furthermore, none of the analyzed subscales of trauma (emotional, sexual, bodily) significantly moderated the serial mediation relationship between ADHD and PLEs (see Table 4). When gender and age were added to these models as covariates, they were not significant predictors in any of them. 

## 4. Discussion

The primary objective of this study was to investigate the relationship between Attention-Deficit/Hyperactivity Disorder (ADHD) symptoms and psychotic-like experiences (PLEs) in a non-clinical sample. The results revealed a significant total effect of ADHD symptoms on PLEs, thus supporting **H1**. Moreover, the results confirm **H2**, namely that there is an indirect pathway between ADHD symptoms and PLEs. Additionally, we identified two key processes that link ADHD and PLEs: increased rumination and heightened negative affect. Collectively, these two processes explained 49.5% of the individual differences in the pathway from ADHD symptoms to PLEs. Finally, the findings of the moderated serial mediation analysis (**H3**) indicated that traumatic experiences do not exert an influence on the tested pathway from ADHD to PLEs. Therefore, **H3** was not confirmed.

The findings of this study are consistent with the high prevalence of ruminative thoughts observed in individuals with ADHD (e.g., [92]) and the detrimental impact of rumination on psychological well-being [93]. This present study demonstrates that ruminative thoughts and negative emotions serve as mediators for the majority of the relationship between ADHD symptoms and PLEs. Although mind wandering is a common phenomenon, there is evidence that prolonged rumination may be associated with the experience of negative emotions [94]. It is noteworthy that in individuals presenting with ADHD symptoms, rumination may serve as a distinctive symptom that prospectively indicates functional impairment [95]. Furthermore, given the high prevalence of ADHD and psychotic disorders [96] and the robust association between ADHD and ruminative thoughts, which predict delusional and hallucinatory experiences [97], our study suggests that ruminative thoughts and the experience of negative affect are important elements in the context of diagnosing and as a treatment focus for ADHD and PLEs symptoms. Furthermore, PLEs, as a part of the psychotic disorder continuum, are most closely related conceptually to a general factor or vulnerability for psychopathology (see [98]), and the affective dynamics are a key factor in virtually all mental health problems [99,100]. 

It is also noteworthy that the majority of participants in this study were young women between the ages of 18 and 35, representing 72.3% of the total sample. The results of Student’s *t*-test indicated that, when compared to men, women in the study group exhibited a significantly higher frequency of ruminative thoughts and negative emotions. A substantial body of literature on this topic (e.g., [101,102,103]) indicates that women are significantly more likely than men to report higher levels of repetitive negative thinking, including ruminative thoughts, particularly at a young age [104]. Therefore, our results corroborate prior findings of elevated levels of ruminative thoughts and, consequently, negative emotions in a cohort of young women, underscoring the significance of these factors in the diagnosis of ADHD and PLEs. However, there is still a need for further data on the specific factors that are associated with an increased risk of mental illness. Therefore, the role of rumination and negative emotions as mediators between ADHD and PLEs may align with the perspective of transdiagnostic vulnerability and should be considered in future studies.

The objective of the second stage of analysis was to examine the potential influence of traumatic experiences on the pathway connecting ADHD symptoms to PLEs through ruminations and NA. The results of the moderated serial mediation analysis did not support the proposed hypothesis (**H3**). Moreover, none of the three subscales of trauma, including emotional, sexual, or bodily traumatic experiences, moderated the hypothesized model. Nevertheless, the findings of this study indicated that trauma continues to exert a considerable influence on the relationship under examination. The results of the correlation analysis demonstrated a relationship between all studied variables (PLEs, ADHD symptoms, ruminations, NA, trauma, and the subscales). Furthermore, our results indicated significant gender differences in the experience of trauma. Specifically, women indicated a significantly higher total trauma score and, notably, a significantly higher score for sexual trauma. This is in line with previous research, showing that women are more likely to experience high-impact traumas with direct life-threatening exposure, such as sexual trauma [105]. It is noteworthy that, based on the M.I.N.I. interview, 27% of individuals in this current study may meet the preliminary criteria for PTSD. A review of the literature on the experience of trauma consistently identifies it as a factor that increases the risk of future PLEs [106,107]. Therefore, while traumatic experiences may not be explicitly represented in our tested models, they serve to increase the risk of developing psychiatric disorders [108,109].

Furthermore, numerous studies (e.g., [110,111,112]) have demonstrated a correlation between trauma and ADHD. This is primarily due to the fact that the cognitive and emotional disturbances that occur in response to experienced trauma (e.g., difficulty concentrating, irritability, high arousal) may overlap with or exacerbate ADHD symptoms (e.g., [113,114]). Furthermore, ADHD symptoms are frequently linked to an elevated probability of exposure to traumatic experiences, particularly during early childhood [115]. Consequently, although ADHD and trauma are discrete domains, their co-occurrence in clinical samples is relatively common, with prevalence rates ranging from 10% to 33% [116]. It is also noteworthy that both disorders are characterized by the experience of intrusive, ruminative thoughts (e.g., [45,117]) and the experience of negative emotions (e.g., [118,119]). It is, therefore, recommended that trauma be considered in future research as a topic for further exploration. 

Research on PLEs strongly benefits from the experience sampling method (ESM) as it allows for real-time data collection, thereby capturing the full range of variability and reducing the impact of retrospective or distorted memories on the data [120]. Prior studies demonstrated the efficacy of the ESM method in accurately capturing momentary fluctuations in psychotic and/or emotional states, which is crucial for elucidating the dynamics of these variables in everyday life (e.g., [121,122,123]). Despite the aggregation of our ESM measures to a weekly average, the averaged results from real-time data provide a richer, more precise, and contextually valid dataset than retrospective data (e.g., retrospective questionnaires) and allow for the identification of patterns that may not be apparent with more traditional measures. Although ADHD symptoms and trauma were not assessed with the ESM, they remained significant factors in the relationships examined. Indeed, there is a clear relationship between negative affect lability and the manifestation of ADHD symptoms [124]. Moreover, some studies suggest that emotional lability may be associated with an increased risk of developing additional mental health problems, including ADHD symptoms [125,126]. Therefore, it is recommended that future research also considers ADHD symptoms in real-time to capture their individual dynamics and their potential impact on other mental health factors. 

## 5. Limitations

Nonetheless, a number of factors should be considered in the interpretation of the results. It is possible that there is a degree of overlap between the symptoms of the two diagnostic categories, namely ADHD and PTSD. The potential for symptom overlap may complicate the ability to discern the precise influence (in this case, the moderating role) of trauma in the relationship between ADHD symptoms, trauma, and PLEs. This could result in the masking or overlapping of results. A traumatic event is one of the most common emerging risk factors for future psychosis [127], and individuals in a (pre-) psychotic state exhibit symptoms such as high impulsivity and frustration or deficits in executive functioning that overlap with clinical symptoms of ADHD [128]. Consequently, diagnoses of ADHD, trauma, and psychosis (or PLEs) should incorporate an evaluation of the other disorders as a standard procedure, and clinicians should be mindful of the potential for these conditions to co-occur or to even function as one syndrome [129]. Such an approach can facilitate the delivery of comprehensive and accurate diagnoses and treatments. 

Another consideration is that the traumatic experience scale (TEC) may not adequately capture the full range of traumatic experiences, particularly regarding their diverse forms (emotional, sexual, and bodily), and showed low reliability. Thus, our study could be replicated using a different or stronger trauma instrument. Furthermore, the TEC is a self-report scale. It is, therefore, possible that individuals reporting such experiences may have understated the significance of the traumatic events in question when completing this questionnaire. One of the coping mechanisms employed in response to traumatic events is the utilization of cognitive avoidance, which is evidenced by the suppression of thoughts and memories, rumination, and dissociation [130]. 

Additionally, the scale we used to measure ADHD symptoms is also a self-report scale based on the criteria for ADHD diagnosis in the DSM-IV [131]. While the revisions to the ADHD diagnosis in the current edition of the DSM-V [36] are subtle, they are more aligned with the current understanding of the disorder’s nature. It is noteworthy that the majority of individuals in our study group exhibited relatively low levels of ADHD symptoms, particularly when assessed using part A of the ASRS questionnaire, which has been identified as the most reliable predictor of ADHD symptoms. Moreover, the majority of the sample consisted of adult women, and the diagnostic criteria for ADHD are still not particularly effective in detecting ADHD in women [132]. The presentation of ADHD symptoms differs significantly between women and men, with women exhibiting greater difficulties with inhibition and cognitive flexibility and men displaying more symptoms of hyperactivity [133]. Additionally, women face challenges in receiving an accurate diagnosis, largely due to the more subtle nature of symptoms (i.e., with less overt hyperactivity) and the potential for misdiagnosis of emotional disturbance [134]. Therefore, further research, with particular regard to the potential influence of gender on the diagnostic process, is warranted.

This present study focused on between-person effects, which was the principal objective. However, this approach also constrains our ability to fully comprehend the precise dynamics of within-person change [135]. Therefore, it would be beneficial for future studies to include real-time intrapersonal measures, particularly for ADHD symptoms and trauma, in order to more accurately capture individual variation and between-person differences in dynamics. It is also important to acknowledge the cross-sectional nature of this study, which constrains our capacity to ascertain the causal mechanisms underlying the relationship between ADHD and PLEs and to note that the findings of this study should not be generalized to the general population, as the majority of the participants were female and did not meet the criteria for a clinical diagnosis.

## 6. Conclusions

In conclusion, the findings of this study underscore the significant role of rumination and negative affect in the relationship between ADHD symptoms and PLEs. By identifying this association, this study provides important findings regarding the complex processes that may contribute to the development of PLEs in individuals with ADHD symptoms. This study is the first to examine this particular relationship. However, given the relative novelty of these findings, further investigation is required to gain a deeper understanding of the underlying mechanisms and potential pathways of influence. It would be beneficial for further research to aim to replicate these results in diverse populations and assess the potential clinical implications for interventions targeting rumination and affect regulation in individuals with ADHD and/or PLEs symptoms.

## Figures and Tables

**Table 1:** Concepts used in this present study and their definitions.

Domain	#	Scale	Items	CA
PLEs	8			0.85
Hallucination-like	3	1–7	“My thoughts are so strong that I can almost hear them”. “I hear things that aren’t really there”. “I see things that aren’t really there”.	0.66
Delusion-like	5	1–7	“I have the sense that some person or force is around me, although I can’t see anyone”. “I see special meanings in advertisements, shop windows, or in the way things are arranged around me”. “I am confused whether something I experienced was real or imaginary”. “My thoughts are influenced by others”. “I can’t get these thoughts out of my head”.	0.78
Ruminative thought	1	1–7	“At the moment, I feel that I am stuck on negative thoughts and can’t get away from them”.	-
Negative affect	5	1–7	“I feel anxious”. “I feel down”. “I feel lonely”. “I feel insecure”. “I feel annoyed”.	0.86

Note. All scales ran from “not at all” (1) to “very much” (7). # = number of items. CA = Cronbach’s alpha. PLEs = Psychotic-like experiences, which comprise hallucinations and delusions experiences.

**Table 2:** Descriptives.

Variable		N	%	Mean	SD	Min	Max	Range
Group	Experimental	99	52.7%					
	Control	89	47.3%					
Gender	Men	52	27.7%					
	Women	136	72.3%					
Age				25.21	5.18	18	35	18–35
Education level								
Primary		6	3.2%					
Secondary		79	42%					
Vocational		1	0.5%					
Higher		102	54.3%					
ADHD (ASRS total) ^1^				34.05	14.36	0	65	0–72
Part A ^2^				10.80	5.37	0	23	0–24
Part B ^3^				23.24	9.88	0	46	0–48
Psychotic experiences ^4,5^				11.92	5.58	6.48	36.36	
Rumination ^4,6^				2.47	1.15	1	6.15	
Negative affect ^4,7^				9.64	4.39	4.20	23.20	
Trauma measurement (TEC total) ^8^				4.98	3.85	0	17	0–29
Emotional trauma ^9^				1.85	1.61	0	6	0–6
Sexual trauma ^9^				0.37	0.65	0	2	0–6
Bodily threat ^9^				0.96	1.05	0	5	0–6

Note. ^1^ ADHD was assessed with the Adult Self-Report (ASRS). ^2^ Part A = Predictive list of ADHD symptoms (ASRS). ^3^ Part B = Control list of ADHD symptoms (ASRS). ^4^ As assessed with the experience sampling method (ESM), see the Methods section for details. ^5^ The average of Psychotic-like experiences (PLEs) over one week. ^6^ The average of rumination scores over one week. ^7^ The average of negative affect (NA) scores over one week. ^8^ Trauma measurement was assessed with the Traumatic Experiences Checklist (TEC). ^9^ Trauma measurement (TEC) subscales. N = Number of participants. SD = Standard deviation. Min = Minimum. Max = Maximum.

**Table 3:** Correlations between study variables (N = 188).

#	Variable	Mean	SD	1.	2.	3.	4.	5.	6.	7.
1.	PLEs ^1^	11.92	5.58							
2.	ADHD ^2^	34.05	14.36	0.41						
3.	Ruminations ^3^	2.47	1.15	0.67	0.53					
4.	NA ^4^	9.64	4.39	0.69	0.56	0.89				
5.	Trauma ^5^	4.98	3.85	0.37	0.31	0.33	0.30			
6.	Emotional trauma ^6^	1.85	1.61	0.33	0.36	0.36	0.35	0.84		
7.	Sexual trauma ^7^	0.37	0.65	0.36	0.23	0.27	0.31	0.60	0.41	
8.	Bodily threat ^8^	0.96	1.05	0.26	0.24	0.26	0.19	0.75	0.51	0.34

Note. All correlations were significant at *p* < 0.001. N = Number of participants. SD = Standard deviation. ^1^ PLEs = Psychotic-like experiences (assessed with the 1-week aggregated ESM data). ^2^ ADHD = Attention-Deficit/Hyperactivity Disorder (assessed with the Adult ADHD Self-Report Scale; ASRS). ^3^ Ruminations (assessed with the 1-week aggregated ESM data). ^4^ NA = Negative affect (assessed with the 1-week aggregated ESM data). ^5^ Trauma experience is treated as a total score from TEC, and ^6,7,8^ are treated as subscales of trauma: ^6^ Emotional trauma captures emotional neglect and/or emotional abuse; ^7^ Sexual trauma captures sexual harassment and/or sexual abuse; ^8^ Bodily threat captures physical abuse in various social settings and intentional threats to one’s life, bizarre punishment, or intense pain.

**Table 4:** Moderated mediation model (Model 85) results.

Moderator (W)	Index of Moderated Mediation	S.E.	CI Lower 95%	CI Upper 95%
Trauma total	0.01	0.02	−0.03	0.05
Emotional trauma	0.01	0.02	−0.03	0.05
Sexual trauma	−0.02	0.03	−0.08	0.02
Bodily threat	−0.02	0.03	−0.08	0.03

Note. S.E. = Standard error. CI = Confidence interval.

## Data Availability

Supporting data findings from this present study are available in the Open Science Framework (OSF) Database (file name: ADHD_PLEs_mediation_moderation_data.sav): https://osf.io/8ju6b/.

## Supplementary Materials

The following supporting information can be downloaded at: https://www.mdpi.com/article/10.3390/jcm13226727/s1, Supplementary Table S1. M.I.N.I. diagnosis. Supplementary Table S2. CAARMS positive symptoms scores. Supplementary Table S3. ESM evaluation not included in the current study. Supplementary Table S4. Variance Inflation Factors. Supplementary Table S5. Condition Index. Supplementary Table S6. Descriptive characteristics of the gender subgroups M (SD). Supplementary Table S7. Descriptive characteristics of the study subgroups M (SD).
