# Supplementary material for: Psychotic-like Experiences and Underlying Mechanisms: An Integrative Model of ADHD Symptoms, Rumination, Negative Affect, and Trauma Experience
Source: J Clin Med. 2024 Nov 8;13(22):6727. doi: 10.3390/jcm13226727 (PMC11594572; doi:10.3390/jcm13226727)
Supplement: Supplementary file 1 [file jcm-13-06727-s001.zip › jcm-3232888-supplementary.pdf]

Supplementary Table S1. M.I.N.I. diagnosis

Variable	N	%
ADHD[1]	25	13.3
Depression	73	38.8
Mania	15	8
OCD[2]	29	15.4
Anxiety	67	35.6
GAD[3]	33	17.6
AUD[4]	6	3.2
SUD[5]	9	4.8
Agoraphobia	34	18.1
Social phobia	49	26.1
Specific phobia	31	16.5
PTSD[6]	52	27.7
PMDD[7]	34	18.1

Note. [1]**ADHD**=Attention-Deficit/Hyperactivity Disorder. [2]**OCD**=Obsessive-Compulsive Disorder. [3]**GAD**=Generalized Anxiety Disorder. [4]**AUD**=Lifetime Alcohol Use Disorder. [5]**SUD**=Lifetime Substance Use Disorder. [6]**PTSD**=Posttraumatic Stress Disorder. [7]**PMDD**=PreMenstrual Dysphoric Disorder. **N**=number of participants.

Supplementary Table S2. CAARMS positive symptoms scores

Domain	Max	Min	Mean	SD
Non-bizarre Ideas	6	0	1.12	1.37
Perceptual Abnormalities	10	0	1.82	2.39
Unusual Thought Content	5	0	0.62	1.10
Disorganized Speech	9	0	1.92	2.69

Note. **Max**=Maximum. **Min**=Minimum. **SD** = Standard Deviation.

Supplementary Table S3. ESM evaluation not included in the current study

Domain	#	Items	Scale
Sleep (quantity)	1	"How many hours did you sleep last night?" (Time from falling asleep to waking up - in hours)	-
Sleep (quality)	1	"How would you describe your sleep today?"	1-7

Stress event	1	"Think of the most important event that has happened to you in the last two hours. The event was for me:"	1-7
Food, drinks	3	"In the past two hours, have you consumed caffeine and/or smoked cigarettes and/or used e-cigarettes/tobacco products?"<br>"Have you had a meal and/or non-alcoholic drink in the past two hours?"<br>"Have you consumed any alcohol in the past two hours?"	1-2
Body image	1	"Evaluate your feelings about the appearance of your body right now:"	1-7*
Activity-related stress	3	"I would rather do something else right now."<br>"What I am doing now is difficult for me."<br>"I enjoy what I am doing."	1-7
Social stress	1	"Who are you currently staying with?"	1-6
Social stress	2	"Right now, I would rather be alone."<br>"At the moment, I find it pleasant to be with these people."<br>***OR***<br>"Right now, I would prefer to be with someone."<br>"Right now, I like being alone."	1-7
Outsider status	1	"Right now, I feel like an outsider."	1-7
Helplessness	2	"I feel upset/nervous because something unexpected has happened."<br>"I feel that important things in my life are out of my control."	1-7
Self-efficacy	2	"I feel confident that I can handle personal problems."<br>"I feel that things are going my way."	1-7
Area-related stress	1	"I find it unpleasant to stay in the present place."	1-7
Aberrant salience	3	"Everything attracts my attention now."<br>"Everything seems to matter now."<br>"I notice things now that I didn't notice before."	1-7
Threat anticipation	3	"Right now, I think something unpleasant is going to happen."<br>"Right now, I am being cautious about protecting myself."<br>"Right now, I am paying attention to details instead of the overall picture."	1-7

Note. Scales which ran from **1-7** "not at all" (**1**) to "very much" (**7**); "very unpleasant" (**1**)* "very pleasant" (**2**)*; Scales which ran from **1-2** "yes" (**1**) to "no" (**2**); Scales which ran from **1-6** (**1**) Alone (**2**) With family (**3**) With partner/husband/wife (**4**) With a friend(s) (**5**) With strangers (**6**) With co-workers/with classmates. #=number of items.

Supplementary Table S4. Variance Inflation Factors

**Variable**	**Tolerance**	**VIF**
Ruminations	.21	4.79
NA[1]	.21	4.75
Trauma[2]	.29	3.48

Emotional trauma[3]	.28	3.56

Note. VIF=Variance Inflation Factor. [1]**NA**=Negative Affect. [2]Trauma is treated as a total score from **TEC**, and [3]Emotiona trauma is treated as a subscale of trauma.

Supplementary Table S5. Condition Index

**Dimension**	**CI**
1	1.0
2	1.46
3	1.59
4	3.97
5	4.81

Note. CI=Condition Index.

Supplementary Table S6. Descriptive characteristics of the gender subgroups M (SD)

**Variable**	**Female (N = 136)**	**Male (N = 52)**	**Group comparison**
Age	25.19 (5.15)	25.25 (5.31)	n.s.
ADHD (ASRS total)[1]	35.12 (14.57)	31.23 (13.54)	n.s.
Part A[2]	11.07 (5.30)	10.12 (5.54)	n.s.
Part B[3]	24.06 (10.16)	21.12 (8.86)	n.s.
Psychotic experiences[4,5]	12.31 (5.87)	10.91 (4.66)	n.s.
Rumination[4,6]	5.60 (1.13)	2.16 (1.14)	$p < 0.05$, $d = 0.39$, **F > M**
Negative Affect[4,7]	10.18 (4.43)	8.22 (3.98)	$p < 0.05$, $d = 0.46$, **F > M**
Trauma measurement (TEC total)[8]	5.35 (4.01)	4.02 (3.22)	$p < 0.05$, $d = 0.35$, **F > M**
Emotional trauma[9]	1.99 (1.66)	1.50 (1.44)	n.s.
Sexual trauma[9]	0.49 (0.72)	0.08 (0.27)	$p < 0.001$, $d = 0.65$, **F > M**
Bodily threat[9]	0.98 (1.06)	0.92 (1.03)	n.s.

Note. [1]ADHD was assessed with the Adult Self-Report (ASRS). [2]Part A=predictive list of ADHD symptoms (ASRS). [3]Part B=control list of ADHD symptoms (ASRS). [4]As assessed with the experience sampling method (ESM), see the method section for details. [5]The average of Psychotic-like Experiences (PLEs) over one week. [6]The average of rumination scores over one week. [7]The average of negative affect (NA) scores over one week. [8]Trauma measurement was assessed with the Traumatic Experiences Checklist (TEC). [9]Trauma measurement (TEC) subscales. **N**=number of participants. **SD**=standard deviation. **F**=Female. **M**=Male.

Supplementary Table S7. Descriptive characteristics of the study subgroups M (SD)

**Variable**	**Experimental group (N = 99)**	**Control group (N = 89)**	**Group comparison**
Gender	1.77 (0.42)	1.67 (0.47)	n.s.
Age	23.92 (4.96)	26.64 (5.07)	$p < 0.001$, $d = -0.54$, **E < C**
ADHD (ASRS total)[1]	43.03 (9.51)	24.06 (12.08)	$p < 0.001$, $d = 1.76$, **E > C**
Part A[2]	13.72 (4.14)	7.56 (4.67)	$p < 0.001$, $d = 1.40$, **E > C**
Part B[3]	29.31 (6.79)	16.49 (8.29)	$p < 0.001$, $d = 1.70$, **E > C**
Psychotic experiences[4,5]	14.94 (5.79)	8.56 (2.68)	$p < 0.001$, $d = 1.39$, **E > C**
Rumination[4,6]	3.05 (1.08)	1.84 (0.86)	$p < 0.001$, $d = 1.23$, **E > C**
Negative Affect[4,7]	12.14 (4.02)	6.85 (2.83)	$p < 0.001$, $d = 1.51$, **E > C**
Trauma measurement (TEC total)[8]	6.55 (4.08)	3.24 (2.67)	$p < 0.001$, $d = 0.95$, **E > C**
Emotional trauma[9]	2.51 (1.69)	1.12 (1.14)	$p < 0.001$, $d = 0.95$, **E > C**

Sexual trauma[9]	0.58 (0.77)	0.15 (0.39)	$p < 0.001$, $d = 0.69$, **E > C**
Bodily threat[9]	1.21 (1.18)	0.69 (0.81)	$p < 0.001$, $d = 0.52$, **E > C**

Note. [1]ADHD was assessed with the Adult Self-Report (ASRS). [2]Part A=predictive list of ADHD symptoms (ASRS). [3]Part B=control list of ADHD symptoms (ASRS). [4]As assessed with the experience sampling method (ESM), see the method section for details. [5]The average of Psychotic-like Experiences (PLEs) over one week. [6]The average of rumination scores over one week. [7]The average of negative affect (NA) scores over one week. [8]Trauma measurement was assessed with the Traumatic Experiences Checklist (TEC). [9]Trauma measurement (TEC) subscales. **N**=number of participants. **SD**=standard deviation. **E**=Experimental group. **C**=Control group.
